# Effects of hibernation on two important contractile tissues in tibetan frogs, *Nanorana parkeri*: a perspective from transcriptomics and metabolomics approaches

**DOI:** 10.1186/s12864-024-10357-4

**Published:** 2024-05-08

**Authors:** Yonggang Niu, Xuejing Zhang, Shengkang Men, Tisen Xu, Haiying Zhang, Xiangyong Li, Kenneth B. Storey, Qiang Chen

**Affiliations:** 1 School of Life Sciences, Dezhou University, Dezhou, 253023 Shandong China; 2 School of Life Sciences, Lanzhou University, Lanzhou, 730000 Gansu China; 3 Department of Biology, Carleton University, Ottawa, ON K1S 5B6 Canada

**Keywords:** *Nanorana parkeri*, Hibernation, Transcriptomics, Metabolomics, Muscle contraction, Metabolism

## Abstract

**Background:**

In response to seasonal cold and food shortage, the Xizang plateau frogs, *Nanorana parkeri* (Anura: Dicroglossidae), enter a reversible hypometabolic state where heart rate and oxygen consumption in skeletal muscle are strongly suppressed. However, the effect of winter hibernation on gene expression and metabolic profiling in these two tissues remains unknown. In the present study, we conducted transcriptomic and metabolomic analyses of heart and skeletal muscle from summer- and winter-collected *N. parkeri* to explore mechanisms involved in seasonal hibernation.

**Results:**

We identified 2407 differentially expressed genes (DEGs) in heart and 2938 DEGs in skeletal muscle. Enrichment analysis showed that shared DEGs in both tissues were enriched mainly in translation and metabolic processes. Of these, the expression of genes functionally categorized as “response to stress”, “defense mechanisms”, or “muscle contraction” were particularly associated with hibernation. Metabolomic analysis identified 24 and 22 differentially expressed metabolites (DEMs) in myocardium and skeletal muscle, respectively. In particular, pathway analysis showed that DEMs in myocardium were involved in the pentose phosphate pathway, glycerolipid metabolism, pyruvate metabolism, citrate cycle (TCA cycle), and glycolysis/gluconeogenesis. By contrast, DEMs in skeletal muscle were mainly involved in amino acid metabolism.

**Conclusions:**

In summary, natural adaptations of myocardium and skeletal muscle in hibernating *N. parkeri* involved transcriptional alterations in translation, stress response, protective mechanisms, and muscle contraction processes as well as metabolic remodeling. This study provides new insights into the transcriptional and metabolic adjustments that aid winter survival of high-altitude frogs *N. parkeri*.

**Supplementary Information:**

The online version contains supplementary material available at 10.1186/s12864-024-10357-4.

## Background

Winter dormancy or hibernation is an adaptive survival strategy used by many animal species to cope with seasonal changes in climate and low availability of food, a biological cycle that many amphibian species must undergo [1]. During hibernation, the body temperature of these ectothermic amphibians falls to ambient temperature [2]. However, ectothermic vertebrates can attempt to maximize their fitness via metabolic compensation or metabolic depression strategies in low-temperature environments [3, 4]. Metabolic rate depression associated with hibernation has been well documented in amphibians [4, 5], and shows that these animals can effectively reduce physiological activities (e.g. oxygen consumption and heart rate) and decelerate the depletion of endogenous fuel reserves by virtue of entering a hypometabolic state in order to prolong survival time in winter [6]. In addition to the direct inhibitory effect of low temperature on various biochemical reactions in vivo, metabolic depression is also accomplished by down-regulating multiple intracellular processes such as ion pumps, mitochondrial proton leakage, and RNA and protein synthesis [7]. Heart and skeletal muscle, as two very important contractile tissues for hibernators, also serve altered functions in winter [8]. Specifically, it has been reported that cardiovascular functions are dramatically suppressed in hibernating ectotherms [9], although heart continues to work at a low rate under cold conditions. Skeletal muscle remains largely immobile during hibernation, but must resume full function upon arousal from torpor [10]. Indeed, a growing number of studies have confirmed that muscle atrophy does not occur in hibernating animals [11, 12]. However, there is little transcriptional or metabolic evidence to suggest that physiological changes in heart and skeletal muscle function occur in ectothermic vertebrates during hibernation.

With the rapid development of high-throughput sequencing technologies, transcriptomics and metabolomics have been widely applied to investigate the molecular mechanisms of winter hibernation in animals. For instance, transcriptome analysis of multiple tissues of the Australian central bearded dragons, *Pogona vitticeps*, revealed that the stress response and protective pathways, such as atrophy protective pathways in skeletal muscle, or cardiac hypertrophic processes in heart, were all induced during hibernation [13]. Transcriptomic analysis of the Chinese alligators, *Alligator sinensis*, also showed that changes in the expression of genes participated in maintaining heart and skeletal muscle contraction during the winter [14]. Metabolomics can identify the end products of gene transcription and protein modification, and thus provide a clear metabolic profile for identifying regulatory pathways associated with winter hibernation [15]. For example, our recent metabolomics study found that plasma metabolites involved in energy metabolism, including amino acids and carbohydrates, were significantly down-regulated in hibernating *N. parkeri* [16]. Additionally, multi-omics analysis has also been used to uncover hibernation mechanisms. For instance, integrative multi-omics analysis revealed that pathways including muscle contraction, nutrition absorption and metabolism, urinary excretion, and immunity function were all down-regulated in hibernating Chinese alligators (*A. sinensis*) [17]. Thus, combining transcriptomics and metabolomics analyses can yield information on both gene expression regulation and metabolic profiling.

*Nanorana parkeri* is an endemic frog species in China and is widely distributed on the Tibetan Plateau, ranging from 2,850 to 5,100 m above sea level (a.s.l) [18]. Our previous study showed that high-altitude populations of *N. parkeri* have stronger hematological parameters and antioxidant defenses than low-altitude populations [19]. The Xizang plateau frog, *N. parkeri*, exhibits a clear seasonal cycle of activity/inactivity and retreats to permanent caves as winter approaches. Our previous investigations of *N. parkeri* focused on the physiological ecology of hibernation [20], seasonal energy metabolism [16, 21], oxidative stress levels and antioxidant defense [22], freeze tolerance and underlying mechanisms [23–25], as well as the effects of acute heat exposure on hibernation [26]. We have shown that the prominent feature of winter hibernation in *N. parkeri* is metabolic rate depression accompanied by a significant reduction in heart rate and mitochondrial respiration rate in skeletal muscle [21]. Specifically, winter frogs had 40–41% lower heart rates compared to summer-active frogs. Moreover, at 20 °C, the state III respiration rate of mitochondria in skeletal muscles of winter frogs decreased by 46% (*P* < 0.01) compared to summer frogs [21]. Heart must remain functional under hypometabolic conditions, and skeletal muscle must regain full function upon recovery in the spring so as to quickly engage in foraging and reproductive processes. However, evidence that occurs at the transcriptional and metabolite levels to support depressed heart rate and lower mitochondrial respiration rate in skeletal muscles in order to maintain their respective functions has remained unexplored. Therefore, evaluation of changes in gene expression and metabolites of cardiac and skeletal muscles between active and hibernating frogs could provide valuable information for enhancing our understanding of the molecular mechanisms that regulate hypometabolism and the functional adaptations that deal with wintertime stresses.

In the present study, we hypothesize that genes and pathways associated with energy metabolism, response to stress, defense mechanisms, and muscle contraction undergo hibernation-related changes in *N. parkeri*. To test the hypothesis, transcriptomics and metabolomics approaches were used to explore changes in gene expression and metabolite levels in myocardium and skeletal muscle from summer-active versus winter-dormant *N. parkeri*.

## Methods

### Sample collection

Adult male *N. parkeri* were collected by hand from wetlands in Damxung County (30.28° N, 91.05° E; 4280 m), Xizang, China. Sampling was conducted in summer (mid-July, *n* = 13) and winter (mid-December, *n* = 12). After weighing body mass and measuring body length, frogs were euthanized by decapitation and dissected. Whole heart and femoral skeletal muscle samples were collected immediately, flash-frozen in liquid nitrogen, and stored at -80 °C. After sampling was completed in both seasons, samples were used for transcriptome sequencing and gas chromatography-mass spectrometry (GC-MS)-based metabolomics analysis.

### Transcriptome analysis and RT-qPCR validation

Transcriptome analysis and RT-qPCR validation were performed as reported previously [27]. Total RNA (*n* = 3 for each season) was extracted from heart and muscle tissues using Trizol reagent and its purity was assessed using a NanoDrop 2000 micro spectrophotometer (Thermo Scientific, USA). RNA concentration and integrity were analyzed using an RNA 6000 Nano Kit with an Agilent Bioanalyzer 2100 (Agilent Technologies, CA, USA). Construction of a cDNA library used a conventional process from Annoroad Gene Technology Corporation (Beijing, China), and were further sequenced with Illumina HiSeq 2500 (paired-end 150-bp). Quality control analysis of the raw data from high-throughput sequencing was performed using FASTQC software (http://www.bioinformatics.babraham.ac.uk/projects/fastqc/) to remove those reads containing adapter and poly-N (“N” larger than 10%) as well as low-quality reads (with > 50% of low-quality bases). Clean reads were used to perform a reference-based RNA-Seq analysis on the BMKCloud platform (http://www.biocloud.net/). Six DEGs in heart and 10 DEGs in skeletal muscle were randomly selected to determine mRNA expression using RT-qPCR. Reverse transcription (RT) was conducted using HiScript III RT SuperMix for qPCR (Vazyme, China), and RT-qPCR was performed with TransStart Tip Green qPCR SuperMix (TransGen Biotech, China) using a Bio-Rad CFX (Bio-Rad, USA). Primer sequences were designed using Primer Premier 5.0 and are shown in Supplementary Table 1. Relative expression levels were analyzed using the 2^−ΔΔCt^ method and β-actin was used as an internal control gene [28]. Three technical replicates for each sample and three biological replicates for each group were conducted.

### Metabolite extraction and metabolomics analysis

Metabolite extraction and metabolomics analysis were conducted as reported previously [27]. Frozen heart (10 mg) and skeletal muscle (50 mg) samples (*n* = 10 for summer, *n* = 9 for winter) were added, respectively, to 250 µL or 1000 µL of chloroform/methanol/water solvent (2:5:2 v:v:v containing 5 µg mL^− 1^ of L-norleucine). Samples were then homogenized in an ice bath using a TissueLyser (JX-24, Jingxin, Shanghai) and centrifuged at 12,000 g at 4 °C for 15 min. Supernatants were collected into new centrifuge tubes. Extraction was then repeated by adding 150 µL of ice-cold methanol to the precipitate, and the supernatant was obtained by merging the two extractions. A 10 µL aliquot of internal standard (0.05 mg mL^− 1^ of ^13^C-^15^N-L-isoleucine) was added into 180 µL of supernatant that was then evaporated to dryness under a nitrogen stream. The dry residue was dissolved in 30 µL of methoxyamine hydrochloride (20 mg mL^− 1^) with pyridine and incubated at 37 °C for 90 min, followed by adding 30 µL of N, O-bis (trimethylsilyl) trifluoroacetamide (BSTFA) with 1% trimethylchlorosilane (TMCS) to derivatize at 70 °C for 60 min. Samples were then used for GC-MS metabolomics analysis. Quality control (QC) samples were prepared by pooling equal aliquots of heart and muscle samples from the two seasons, respectively. Metabolomics analysis was conducted using an Agilent 7890 A gas chromatography system coupled to an Agilent 5975 C inert MSD system (Agilent Technologies Inc., CA, USA) following a previously described protocol [29]. Peak picking, alignment, deconvolution, and further processing of raw GC-MS data were performed as reported previously [30]. Metabolite identification was performed by matching mass spectra to an in-house standard library (Profleader Biotech Co., Ltd., Shanghai, China), Golm Metabolome Database, and Agilent Fiehn GC-MS Metabolomics RTL Library.

### Statistical analysis

Transcriptomics data was quantified using StringTie software (v2.2.1) and normalized using FPKM (Fragments Per Kilobase of transcript per Million fragments mapped). Differentially expressed genes were screened using a criterion of |Log_2_FoldChange| ≥ 1 and False Discovery Rate (FDR) < 0.05. Functional enrichment analysis, including Gene Ontology (GO) and Kyoto Encyclopedia of Genes and Genomes (KEGG), was conducted with a threshold q value (adjusted *P* value) < 0.05.

Metabolomics data including sample names, metabolites, variables (rt_mz), and peak abundances were normalized against total peak abundances and then imported into SIMCA software (V14.1, Sweden). Univariate and multivariate statistical analyses, including *t*-test, principal component analysis (PCA), and orthogonal partial least squares discriminant analysis (OPLS-DA), were performed to screen for DEMs between summer and winter. Values of *P* < 0.05 and variable importance for the projection (VIP) > 1 were considered statistically significant. Pathway analysis was conducted using MetaboAnalyst 5.0 (https://www.metaboanalyst.ca/) with criteria of impact scores > 0.05 and -log_10_(*P* value) > 1.0.

## Results

### Transcriptome analysis

After quality control analysis, a total of 50.41Gb and 46.86Gb of clean data were obtained in heart and skeletal muscle, respectively. The values for Q20, Q30, GC content, and map rates are shown in Supplementary Table 2. A total of 2407 DEGs were identified in heart between summer and winter, of which 1321 genes were up-regulated and 1086 genes were down-regulated. In skeletal muscles, there were 2938 DEGs including 1368 up-regulated genes and 1570 down-regulated genes.

**Fig. 1:**
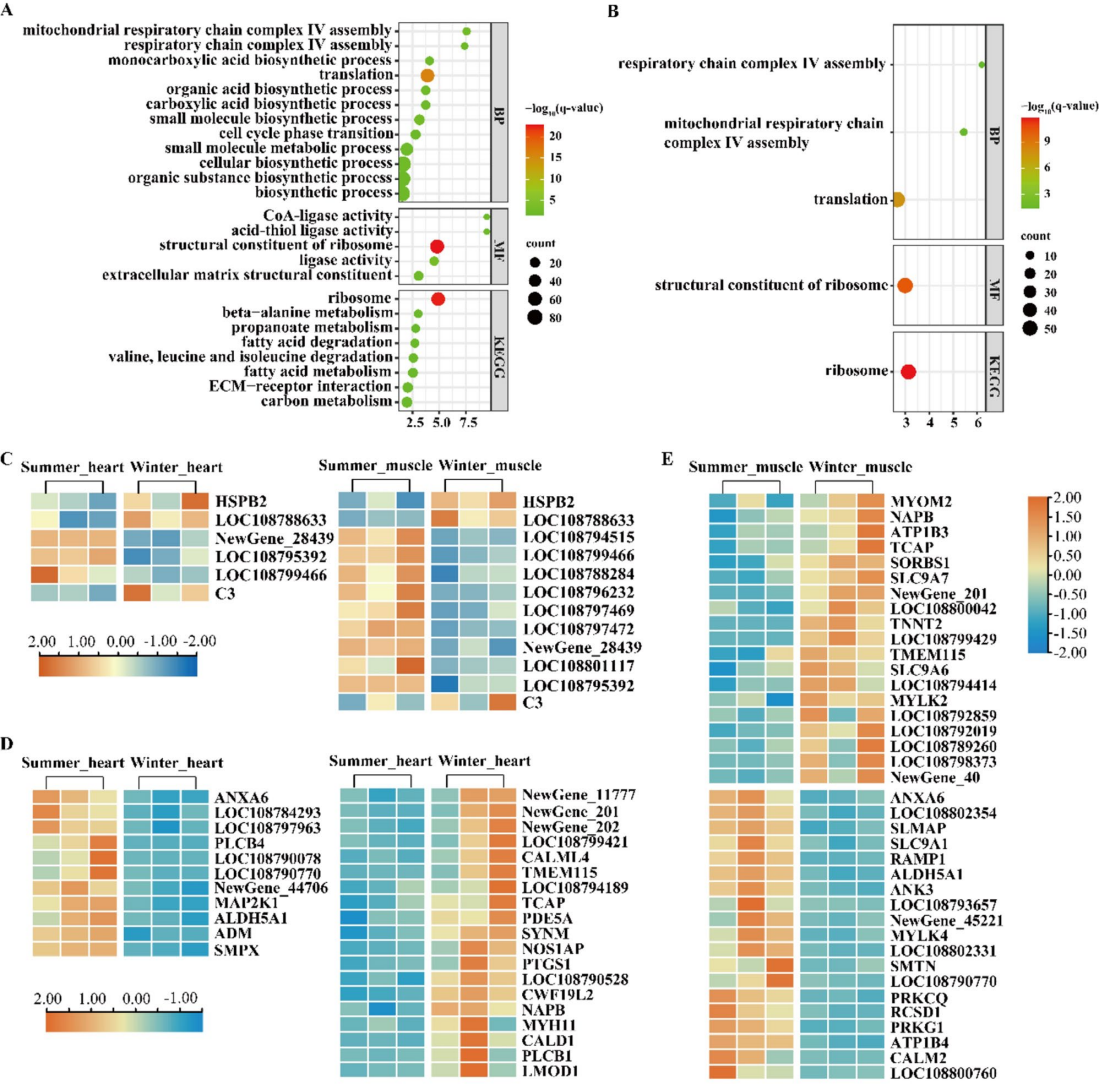
Bubble charts showing GO and KEGG enrichment analysis of differentially expressed genes in heart (**A**) and skeletal muscle (**B**) of *N. parkeri*. The color shades and circle size represent different q values and gene count, respectively. Heat map showing differentially expressed genes encoding protective molecules (**C**) and those involved in muscle contraction in cardiac (**D**) and skeletal muscles (**E**). Red color indicates highly expressed genes, and blue indicates low expressed genes

In heart, GO enrichment analysis showed that DEGs were significantly enriched in translation, small molecule biosynthetic process, mitochondrial respiratory chain complex IV assembly, cellular biosynthetic process, small molecule metabolic process, organic acid biosynthetic process, and cell cycle phase transition in biological process terms (Fig. 1A). Molecular function terms, including structural constituent of ribosome, ligase activity, extracellular matrix structural constituent, CoA-ligase activity, and acid-thiol ligase activity, were all significantly enriched by DEGs (Fig. 1A). KEGG enrichment analysis showed that DEGs in heart were mainly enriched in ribosome, fatty acid metabolism, valine, leucine and isoleucine degradation, fatty acid biosynthesis, ECM-receptor interaction, carbon metabolism, beta-alanine metabolism, and propanoate metabolism (Fig. 1A). In skeletal muscles, GO enrichment analysis showed that DEGs were significantly enriched in translation, mitochondrial respiratory chain complex IV assembly, and respiratory chain complex IV assembly in biological process terms, and structural constituent of ribosome in molecular function terms (Fig. 1B). KEGG enrichment analysis showed that DEGs were enriched in ribosome in skeletal muscle (Fig. 1B).

**Fig. 2:**
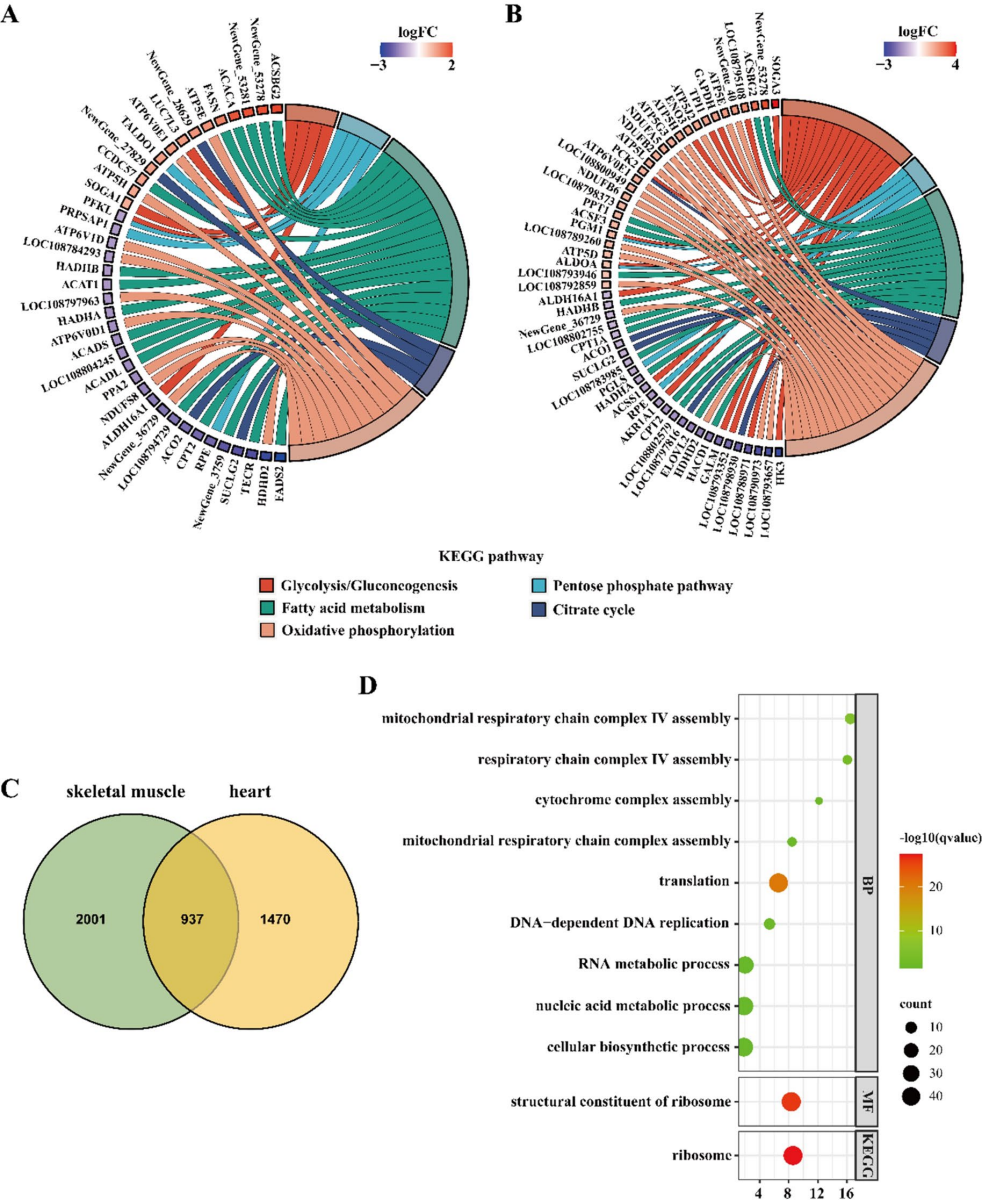
Chord diagrams showing the differentially expressed genes participating in metabolic pathways in heart (**A**) and skeletal muscle (**B**). The size of each node represents the overlapped genes in each term, and the color shades represent the log_2_fold change of each differentially expressed gene. Venn diagram showing the number of differentially expressed genes shared in heart and skeletal muscle (**C**). Bubble chart showing GO and KEGG enrichment analysis of differentially expressed genes shared in heart and skeletal muscle (**D**)

DEGs linked functionally to “response to stress” and “defense mechanisms” are exhibited in Fig. S1 and S2. Changes in the expression of DEGs encoding heat shock proteins (HSPs) and antioxidant enzymes as well as those involved in immune defense functions are shown in Fig. 1C. HSP genes, including *HSPA13*, *HSPB2*, and *LOC108788633*, were all up-regulated in heart, and *LOC108788633* and *HSPB2* were up-regulated in skeletal muscle. The expression of genes *Newgene_28439* and *LOC108795392* encoding glutathione-S-transferase (GST) and *LOC108799466* encoding catalase (CAT) were significantly down-regulated in winter-collected heart. Moreover, the gene *LOC108794515* encoding superoxide dismutase (SOD), *LOC108799466* encoding CAT, and the genes encoding GST including *LOC108788284*, *LOC108796232*, *LOC108797469*, *LOC108797472*, *NewGene_28439*, *LOC108801117*, and *LOC108795392* were all significantly down-regulated in skeletal muscle during the winter. Hibernating frogs had a higher expression of complement component C3 in both heart and skeletal muscle than summer-active frogs. DEGs in cardiac and skeletal muscles involved in muscle contraction are presented in Fig. 1D and E, respectively. In heart, genes *LOC108790078* and *LOC108790770*, encoding dystrophin, were significantly down-regulated, but the gene *MYH11* (encoding myosin heavy chain) was significantly up-regulated in winter (Fig. 1D). In skeletal muscles, the gene *LOC108790770* was significantly down-regulated, but genes *TCAP* (encoding telethonin) and *MYOC* (encoding myocilin) showed significant up-regulation in winter (Fig. 1E). Moreover, DEGs involved in energy metabolic processes in cardiac and skeletal muscles are shown in Fig. 2A and B, respectively, including glycolysis/gluconeogenesis, pentose phosphate pathway, fatty acid metabolism, citrate cycle (TCA cycle), and oxidative phosphorylation.

A total of 937 DEGs were shared in cardiac and skeletal muscles (Fig. 2C). GO enrichment analysis revealed that these DEGs were significantly enriched in translation, metabolic processes in biological process terms, and structural constituent of ribosome in molecular function terms (Fig. 2D). KEGG enrichment analysis showed shared DEGs were significantly enriched in ribosome (Fig. 2D).

**Fig. 3:**
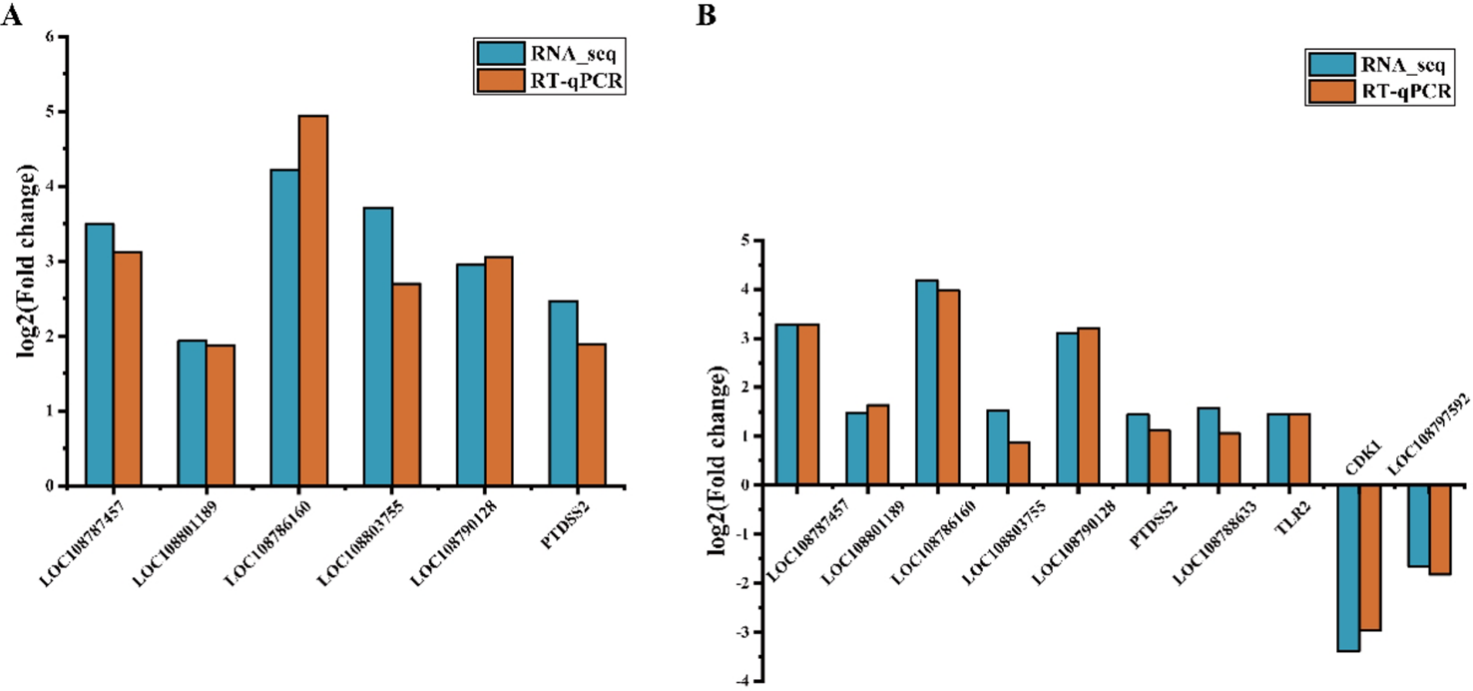
Differentially expressed genes in heart (**A**) and skeletal muscle (**B**) were validated by comparison of RNA-seq and RT-qPCR, respectively

RT-qPCR results showed that expression levels of genes, including *LOC108787457*, *LOC108801189*, *LOC108786160*, *LOC108803755*, *LOC108790128*, and *PTDSS2*, all increased significantly in heart and skeletal muscles during hibernation (Fig. 3A, B). Moreover, both *LOC108788633* and *TLR2* were also significantly up-regulated whereas *CDK1* and *LOC108797592* genes were significantly down-regulated in skeletal muscles (Fig. 3B). These changes in gene expression coincide with findings from RNA-sequencing.

### Metabolome analysis

**Fig. 4:**
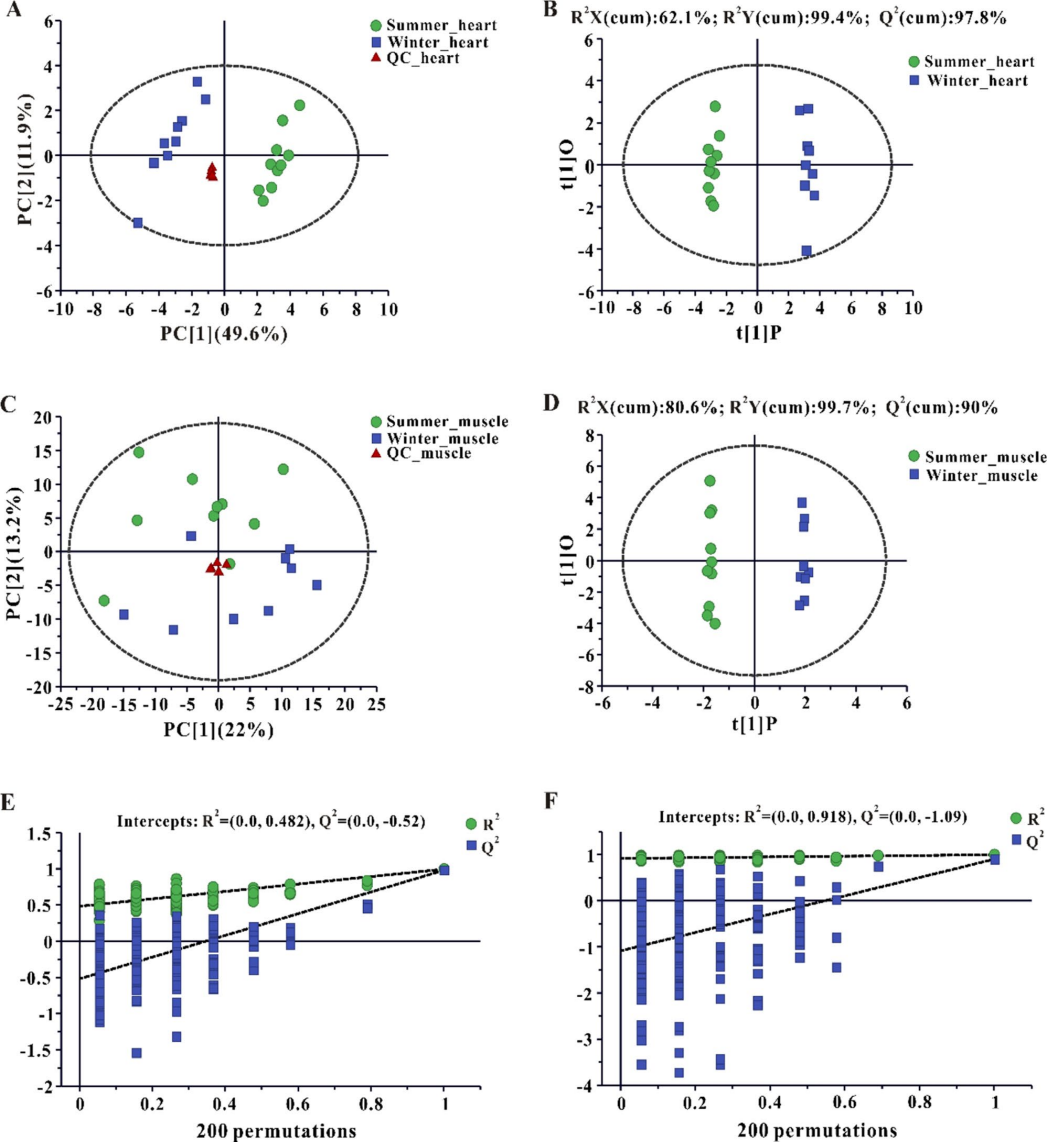
Principal component analysis (PCA) score plots (**A**, **C**), the orthogonal projection to latent structures discriminant analysis (OPLS-DA) scores plots (**B**, **D**), and permutation tests (**E**, **F**). Heart: **A**, **B**, **E** and skeletal muscle: **C**, **D**, **F**, respectively. The three groups are distinguished as summer (green); winter (blue); and Quality Control (QC, red)

**Fig. 5:**
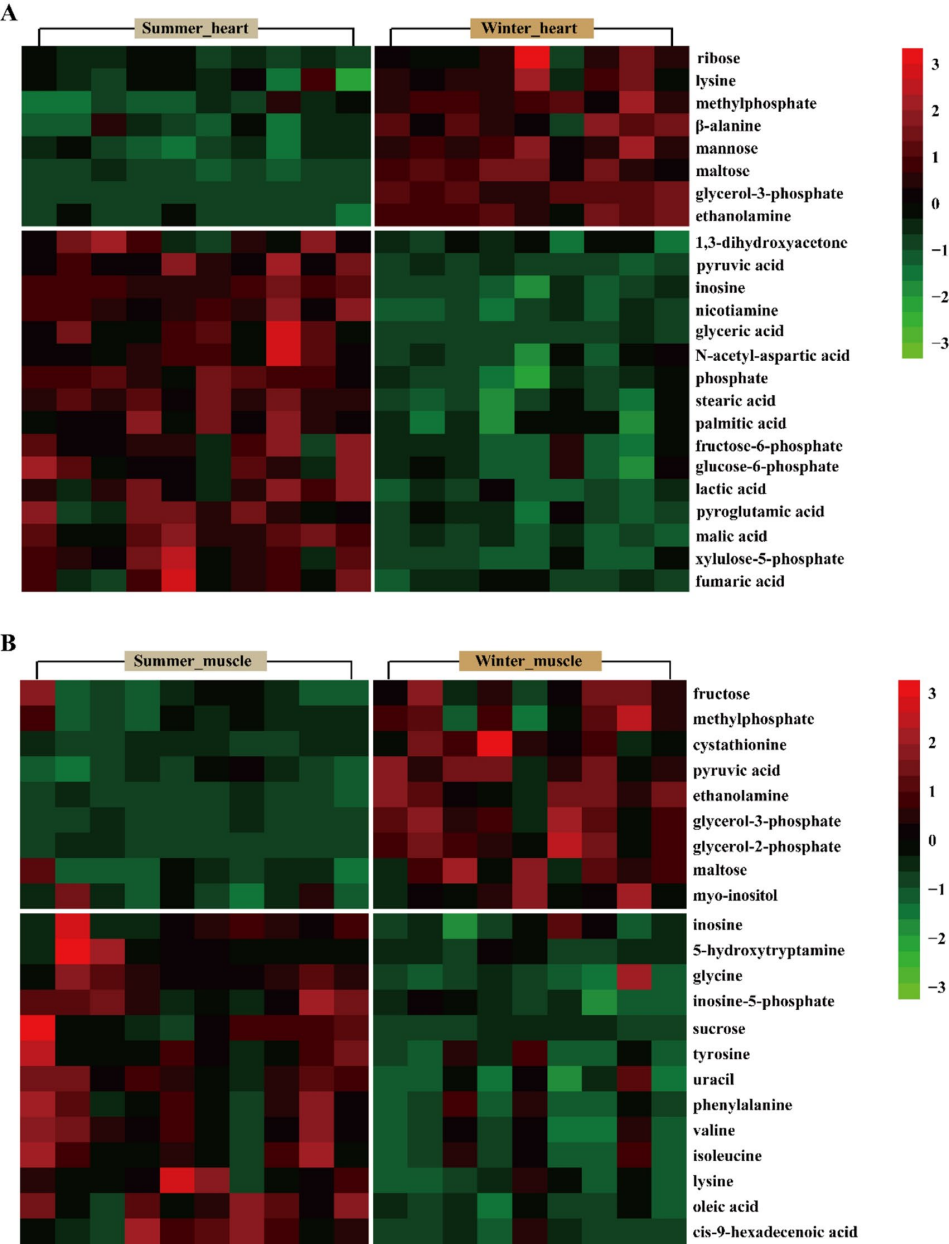
Heat map showing differentially expressed metabolites in heart (**A**) and skeletal muscle (**B**). Red color indicates high-abundance metabolites, and green indicates low-abundance metabolites

**Fig. 6:**
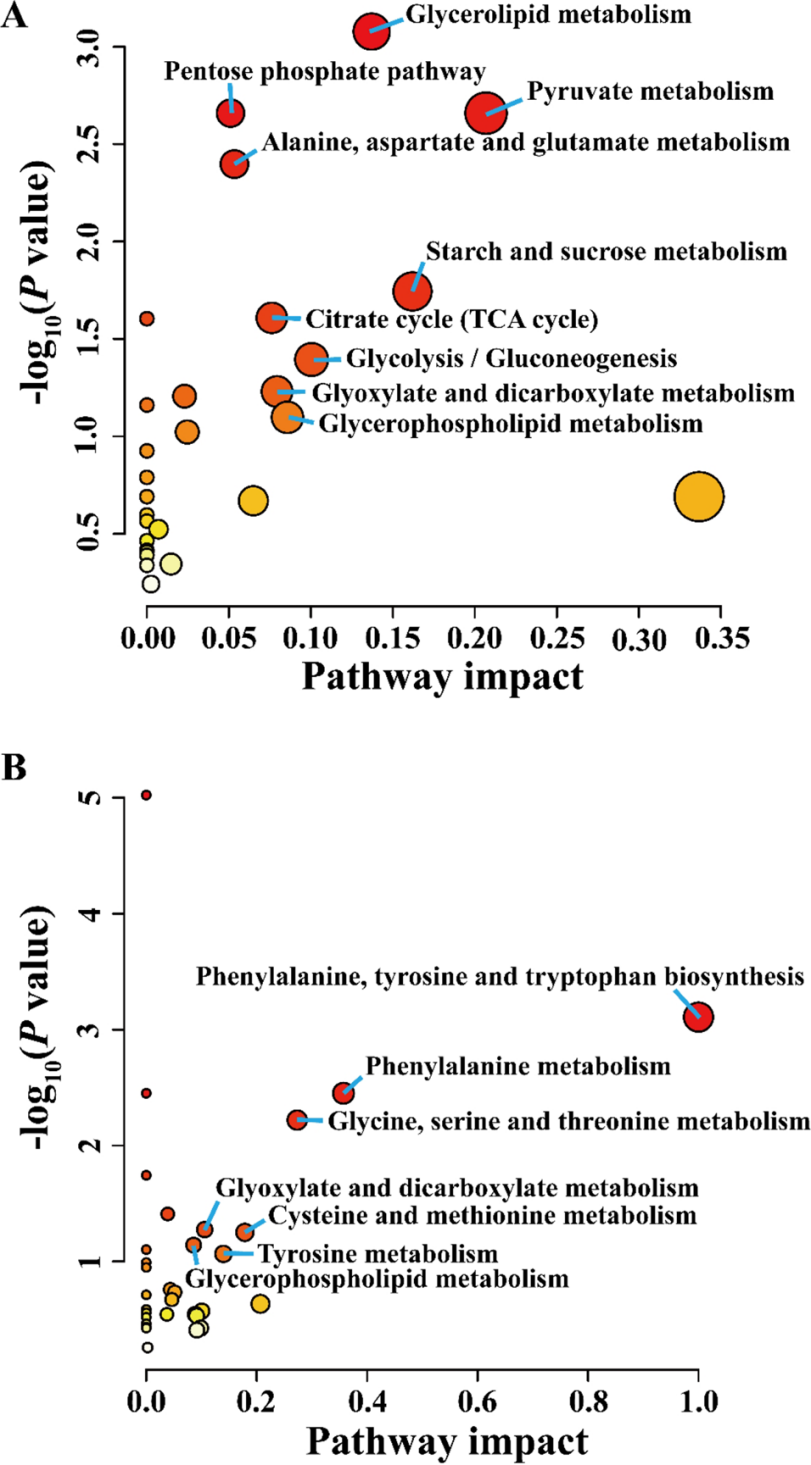
Bubble charts showing pathway analysis of differentially expressed metabolites in heart (**A**) and skeletal muscles (**B**) of *N. parkeri*. The color shades and circle size represent different *P* values and pathway impact values, respectively

PCA and OPLS-DA were visualized as scatter plots (Fig. 4A, B, C, D), and showed a large spread between summer and winter and a good agreement between replicates from each season. The R^2^X, R^2^Y, and Q^2^ of OPLS-DA model in heart were 62.1%, 99.4%, and 97.8%, respectively (Fig. 4B). In skeletal muscles, the values for R^2^X, R^2^Y, and Q^2^ of OPLS-DA model were 80.6%, 99.7%, and 90%, respectively (Fig. 4D). These results showed that OPLS-DA model has a high reliability. Permutation tests showed that the values of R^2^ and Q^2^ were 0.482 and − 0.52 in the heart (Fig. 4E), respectively, with values of 0.918 and − 1.09 in skeletal muscle (Fig. 4F), suggesting that the OPLS-DA model was not over-fitted. A total of 24 DEMs were detected in heart between the two groups, including 8 up-regulated and 16 down-regulated metabolites (Fig. 5A). In skeletal muscles, a total of 22 DEMs were identified, including 9 up-regulated and 13 down-regulated metabolites (Fig. 5B). Pathway analysis showed that DEMs in the heart were significantly enriched in glycerolipid metabolism, pentose phosphate pathway, pyruvate metabolism, alanine, aspartate and glutamate metabolism, starch and sucrose metabolism, TCA cycle, glycolysis/gluconeogenesis, glyoxylate and dicarboxylate metabolism, and glycerophospholipid metabolism (Fig. 6A), whereas DEMs in skeletal muscles were mainly involved in amino acid metabolism (Fig. 6B).

## Discussion

In the present study, we used transcriptomic and metabolomic approaches to obtain a broad overview of gene expression and metabolite changes associated with hibernation in cardiac and skeletal muscles of the high-altitude frog, *N. parkeri*. Transcriptome analysis revealed that genes involved in translation, response to stress, defense mechanisms, muscle contraction, and metabolic processes undergo hibernation-related alterations. Metabolic profiling also showed a significant change in the levels of various intermediates that participate in the process of energy metabolism.

### Effects of hibernation on the expression of genes related to the translation process

Protein synthesis typically accounts for 25–30% of cellular O_2_ consumption and, when suppressed, a reduction of these functions contributes to metabolic rate depression [31]. Here, we found that up-regulated genes in both heart and muscle were significantly enriched in translation. Similarly, our previous results also showed that DEGs in liver were significantly enriched in translation during hibernation [27]. In the hypothalamus of *A. sinensis*, thirteen genes associated with protein synthesis were highly expressed during hibernation [32]. Moreover, in the testis of hibernating Chinese alligators, up-regulated genes were also enriched in GO terms “ribosome” and “translation” [33]. At low temperatures, up-regulation of some genes contributes to enhancing protein expression and thus can compensate for a temperature-related decrease in enzyme activity [34]. Therefore, we consider that increased expression of genes involved in the translation process and ribosomal pathway may be closely related to compensating for the reduced enzyme activity under low-temperature conditions, since the corresponding gene products can stabilize the protein machinery to maintain homeostasis. Moreover, given that protein synthesis is known to be inhibited in overwintering frogs [5], we propose that maintaining a high rate of mRNA translation during winter supports a molecular strategy for rapid recovery of function upon emergence from hibernation, since the cold temperatures on the plateau forces frogs into a relatively brief season of activity, reproduction and growth.

### Effects of hibernation on the expression of genes encoding protective molecules

Heat shock proteins (HSPs) are a class of chaperone proteins and play cytoprotective roles in folding new proteins and refolding damaged proteins in order to maintain protein function under stress conditions [35]. The up-regulation of genes, encoding HSPs, in the heart and skeletal muscle of hibernating *N. parkeri* plays an important role in maintaining protein homeostasis within tissues at low temperatures. Similar to observations in Asiatic toads (*Bufo gargarizans*), HSP genes were up-regulated during torpor [36]. Up-regulation of antioxidant defenses is also considered to be essential for tolerating environmental stresses, such as hypoxia, dehydration, and freezing exposure during hibernation, and responding to rapid ischemia-reperfusion upon arousal in ectothermic vertebrates [37, 38]. However, we previously found that the activities of SOD and CAT were significantly decreased in skeletal muscle during hibernation [22], which was well supported by a lower expression of the gene *LOC108794515* (encoding SOD) and *LOC108799466* (encoding CAT). In both heart and skeletal muscle, GST activity showed no significant difference between summer and winter, and no significant changes were also found in CAT activity in the heart [22]. However, the expression of genes, such as *NewGene_28439* and *LOC108795392* (encoding GST), was significantly lower in both tissues of hibernating frogs, and the gene *LOC108799466* in heart was down-regulated in winter. We speculated that the activities of GST and CAT can be regulated at the post-transcriptional level. For instance, our recent study has demonstrated that phosphorylation levels of GST and CAT increased in liver of hibernating *N. parkeri* [39]. In addition, complement component C3 plays versatile roles in tissue homeostasis, immune surveillance, and adaptive immune regulation [40]. In hibernating bullfrogs (*Rana catesbeiana*), the expression of hepatic complement component C3 showed a significant increase [41]. Therefore, elevated expression of complement component C3 in both heart and muscle of hibernating *N. parkeri* contributes to enhancing immune defenses in winter.

### Effects of hibernation on the expression of genes associated with myocardial contraction

Cardiac muscle contraction is a complex process triggered by Ca^2+^ inward flow that is regulated by a variety of calcium channels in the cell membrane. For instance, in hibernating alligators, many voltage-dependent calcium channel genes were down-regulated, contributing to lowering heart rate [17]. In the present study, we found that only *CACNA1G* (encoding voltage-dependent T-type calcium channel subunit alpha-1G) was down-regulated, suggesting that this gene was crucial for reducing heart rate of hibernating *N. parkeri*. Lower body temperature in overwintering ectotherms typically increases blood viscosity and reduced energy supply that can be extremely challenging for maintaining heart function. Cardiac muscle contraction was also largely determined by the mRNA level of myosin that is a major structural protein of cardiac muscle [42]. For instance, expression of myosin heavy chain (*MHC*) type β was significantly up-regulated in hibernating *Rana bedriagae* as compared to active frogs, which was associated with a lower contractile response of ventricular myocardium to calcium [42]. In this study, a higher expression of *MYH11* (encoding myosin heavy chain) suggested a compensatory mechanism to preserve basic cardiac muscle contraction under stressful conditions, although heart rate was depressed in winter. Dystrophin, found in the cardiomyocyte membrane cytoskeleton, plays an important role in stabilizing the peripheral plasma membrane during the repetitive distortion associated with cardiac contractions [43]. Therefore, down-regulation of dystrophin (*LOC108790078* and *LOC108790770*) may be a potential contributor to the lower heart rate in *N. parkeri* during hibernation.

### Effects of hibernation on the expression of genes associated with muscle atrophy

Telethonin, as a muscle regulating factor, participates in sarcomere assembly [44]. Telethonin deficiency initially manifests as a congenital muscular dystrophy [45]. Therefore, up-regulating *TCAP* (encoding telethonin) in hibernating *N. parkeri* may help prevent muscle atrophy due to prolonged inactivity and starvation in winter. Typically, frogs will engage in mating and reproductive behaviors shortly after emerging from winter hibernation [5], and thus skeletal muscles are unlikely to undergo any dramatic degradation or fibrous-type remodeling when overwintering. Similarly, muscles remain quiescence for months without any sign of muscle atrophy in aestivating frogs, *Cyclorana alboguttata* [46]. Myocilin is a prohypertrophic protein and plays an important role in regulating muscle quality and stabilizing the dystrophin glycoprotein complex and sarcolemma. Maintaining myocilin expression in skeletal muscle significantly attenuated muscle loss, whereas severe muscle atrophy occurred in the absence of myocilin expression [47]. Moreover, myocilin expression is down-regulated or even absent in animal models of myofiber atrophy, sarcoplasmic fragility and impaired muscle regeneration [48]. Therefore, elevated expression of *MYOC* (encoding myocilin) likely contributes to preventing muscle atrophy or lesions in hibernating *N. parkeri*. Dystrophin can also mediate signaling pathways associated with muscle atrophy and hypertrophy, and maintaining dystrophin levels can inhibit substantial muscle loss [49]. Therefore, a lower expression of *LOC108790770* encoding dystrophin in winter supports the lack of atrophy seen in skeletal muscle. Of course, additional evidence, including size (cross-sectional area), strength, and endurance of muscle, needs to be further explored.

### Effects of hibernation on lipid metabolism, glycolysis and TCA cycle

In our study, lower levels of palmitic acid and stearic acid in heart as well as oleic acid and cis-9-hexadecenoic acid in skeletal muscle suggested that lipid metabolism was suppressed in hibernating *N. parkeri*. This is also corroborated by the transcriptome results that the genes (*HADHB* and *HADHA*) encoding 3-hydroxyacyl-CoA dehydrogenase (an indicator of fatty acid utilization capacity) showed a significant down-regulation in the myocardium of hibernating *N. parkeri*. Similarly, lower levels of palmitic acid and stearic acid were also observed in the heart of sand lizards, *Phrynocephalus przewalskii*, in a low-temperature acclimation group (4 °C, 40 days) as compared to animals in a normal temperature group (25 °C, 40 days) [50]. Lower levels of glucose-6-phosphate (G6P), fructose-6-phosphate (F6P), pyruvate, and lactate indicated that glycolysis was depressed in heart during the winter, although DEGs were not significantly enriched in these metabolic pathways. The results suggest that the regulation of enzyme activity in these metabolic pathways may not occur primarily at the transcriptional level, but more likely at the post-transcriptional level. Our recent study has confirmed that reversible protein phosphorylation, as a crucial regulatory mechanism, controlled the activities of various metabolic enzymes [39]. Moreover, lower levels of pyruvate and lactate in the heart of winter-collected frogs is consistent with down-regulated expression of *LDHD* encoding D-lactate dehydrogenase. Similar to findings in turtles, *Trachemys scripta*, fasting and cold-acclimation resulted in lower levels of intermediate metabolites (G6P, F6P, pyruvate, and lactate) in heart [51]. The accumulation of pyruvate in skeletal muscle may be due to a lower pyruvate dehydrogenase (PDH) activity, since this enzyme allows a conversion of pyruvate to acetyl-CoA and its entry into the TCA cycle. This would facilitate a breakdown of carbohydrate stores that should instead be conserved during overwintering [52]. Although the mRNA levels of PDH did not differ significantly in skeletal muscle between the two seasons, the activity of this enzyme complex was regulated by post-translational modifications in the liver [39]. Furthermore, two intermediate metabolites of the TCA cycle, fumaric acid and malic acid, were significantly reduced in winter, suggesting that the TCA cycle was slowed. Similar results were also observed in cold-stressed insects, where fumarate and malate showed significant down-regulation in the cold [53].

### Effects of hibernation on the amino acid metabolism

In the present study, the higher level of alanine in heart is likely derived from pyruvate, rather than other amino acids, consistent with lower levels of pyruvate. Conversion of pyruvate to alanine, instead of lactate, is beneficial because lactate accumulation can cause acidosis whereas alanine, as neutral amino acid, can be preserved in high concentrations and potentially contribute to osmoregulation if frogs experience dehydration or freezing stress during the winter. Similarly, in turtles, *Trachemys scripta*, anoxic exposure induced a significant accumulation of alanine in the heart [51] as this has also been seen in frozen frogs (*Rana sylvatica*) where alanine can play a cryoprotective or osmoregulatory role [54]. However, this remains to be evaluated in hibernating *N. parkeri*. Muscle atrophy is the result of net protein loss being accelerated. In Alaskan frogs, *R. sylvatica*, significant atrophy occurs in skeletal muscles during winter and the degradation of muscle proteins releases large amounts of amino acids with the up-regulated activity of glutamate dehydrogenase [55, 56]. However, no significant change was observed in the gene expression of glutamate dehydrogenase (*LOC108792950*) in skeletal muscle of *N. parkeri*, suggesting that muscle atrophy did not develop in this species during hibernation. This echoes a paradigm of atrophy resistance in burrowing and aquatic frogs [57, 58]. Moreover, the levels of several essential amino acids were significantly decreased in skeletal muscle, which may be due to the cessation of feeding in winter, since these amino acids must be replenished from the diet. Transcriptomics data revealed that most DEGs, such as *ASS1* (encoding argininosuccinate synthase), *ASNS* (encoding asparagine synthetase), and *LOC108802063* (encoding aspartate aminotransferase), that participate in amino acid transport and metabolic processes were significantly down-regulated in winter. These results provide strong evidence that amino acid metabolism was significantly suppressed in skeletal muscle during winter and that muscle atrophy may not be visible.

## Conclusions

To sum up, our findings provide comprehensive evidence for differential gene expression and metabolic profiles in the heart and skeletal muscle of *N. parkeri* under active versus dormant states. Significant changes occurred in the expression of genes related to translational processes and contractile function, which may represent core transcriptional responses in cardiac and skeletal muscles during hibernation. Moreover, we did not detect any transcriptomic or metabolomic signatures of skeletal muscle atrophy in this hibernator. As anticipated, significant down-regulation of carbohydrate metabolism, fatty acid metabolism, and amino acid metabolism were detected, suggesting that overwintering frogs adjust their metabolism to slow down fuel/energy consumption in order to prolong winter survival. Up-regulation of some genes, such as *HSPA13*, *HSPB2*, *LOC108788633*, and *C3*, aid in maintaining protein homeostasis and enhancing protein protection in winter. The present study contributes to elucidating the molecular features behind the adaptation of amphibian species to winter conditions at high altitudes.

### Electronic supplementary material

Below is the link to the electronic supplementary material.

Supplementary Material 1

## Data Availability

Raw transcriptomics data and metabolomic data were deposited in the Genome Sequence Archive (GSA) database (https://ngdc.cncb.ac.cn/gsa/) and OMIX (https://ngdc.cncb.ac.cn/omix) with project number PRJCA021216 and PRJCA021411, respectively.
