# Supplementary material for: Effects of hibernation on two important contractile tissues in tibetan frogs, Nanorana parkeri: a perspective from transcriptomics and metabolomics approaches
Source: BMC Genomics. 2024 May 8;25:454. doi: 10.1186/s12864-024-10357-4 (PMC11080311; doi:10.1186/s12864-024-10357-4)

Supplementary table 1. Primer sequences used in this study

| Genes | Primers Sequence (5’ to 3’) |
| --- | --- |
| LOC108787457(F) | CAGTGACCGTACACCACACA |
| LOC108787457(R) | CAGCTGGCACTACACACTGA |
| LOC108801189(F) | CCTCAGAAACCAGAGCGTG |
| LOC108801189(R) | GTGCAGGTCCAGAAGAGCAT |
| LOC108786160(F) | TGCGAATGTGCAAAGGGATG |
| LOC108786160(R) | ACACTGATCACAGAAGACGATTCA |
| LOC108803755(F) | ATGGTGACGCTGGACTACAC |
| LOC108803755(R) | GGGCAGTAAAAGGGTCCAGT |
| LOC108790128(F) | TGTGGGGAGCCAAGGAGATA |
| LOC108790128(R) | CACCGGGATCACCAGACAAT |
| PTDSS2(F) | GCGGTGTTGGATTCTGGGTA |
| PTDSS2(R) | CTTGTCTCTGCCGACGGATT |
| LOC108788633(F) | TCCCCAACAAACAGGATAGAACC |
| LOC108788633(R) | GGTTCCAGATTTGGCGATGG |
| TLR2(F) | CAGGACTGGATGGAAGGGC |
| TLR2(R) | TGTGGCCAACCTACAAGAAGTT |
| CDK1(F) | ATGGCTCTGGTTGGTGGATG |
| CDK1(R) | GTAGTGATTCCACCTCCGGC |
| LOC108797592(F) | GTGGCGCACACTCAGACTTA |
| LOC108797592(R) | CTCTCCTGTGGCTTGCTTGA |
| actinB(F) | AAGATGATATCGCCGCCCTG |
| actinB(R) | CGACCCACAATGGATGGGAA |

Supplementary table 2. Summary of RNA-seq quality data of heart and skeletal muscles from summer- and winter-collected *N. parkeri*

| Sample ID | Clean reads | Clean bases | GC(%) | Q20(%) | Q30(%) | Map rate(%) |
| --- | --- | --- | --- | --- | --- | --- |
| S_heart_1 | 57,019,214 | 8,552,882,100 | 45.47 | 94.31 | 87.74 | 88.48 |
| S_heart_2 | 52,510,904 | 7,876,635,600 | 45.37 | 94.44 | 87.93 | 88.71 |
| S_heart_3 | 53,442,880 | 8,016,432,000 | 45.33 | 94.58 | 88.15 | 89.42 |
| W_heart_1 | 57,052,104 | 8,557,815,600 | 45.87 | 94.86 | 88.9 | 85.90 |
| W_heart_2 | 54,812,266 | 8,221,839,900 | 44.97 | 94.67 | 88.43 | 86.34 |
| W_heart_3 | 61,210,950 | 9,181,642,500 | 47.36 | 91.39 | 83.76 | 60.38 |
| S_muscle_1 | 48,022,292 | 7,203,343,800 | 47.77 | 96.48 | 91.52 | 90.65 |
| S_muscle_2 | 48,591,260 | 7,288,689,000 | 46.92 | 96.50 | 91.60 | 90.17 |
| S_muscle_3 | 48,159,708 | 7,223,956,200 | 46.96 | 96.38 | 91.37 | 91.27 |
| W_muscle_1 | 53,937,584 | 8,090,637,600 | 48.04 | 95.19 | 89.29 | 85.77 |
| W_muscle_2 | 55,333,838 | 8,300,075,700 | 47.95 | 94.83 | 88.47 | 86.70 |
| W_muscle_3 | 58,365,404 | 8,754,810,600 | 47.48 | 95.43 | 89.77 | 86.95 |

Supplementary Fig. 1. Heat map showing differentially expressed genes in the heart involved in “response to stress” (A) and “defense mechanisms” (B), respectively. Red color indicates highly expressed genes, and blue indicates low expressed genes.


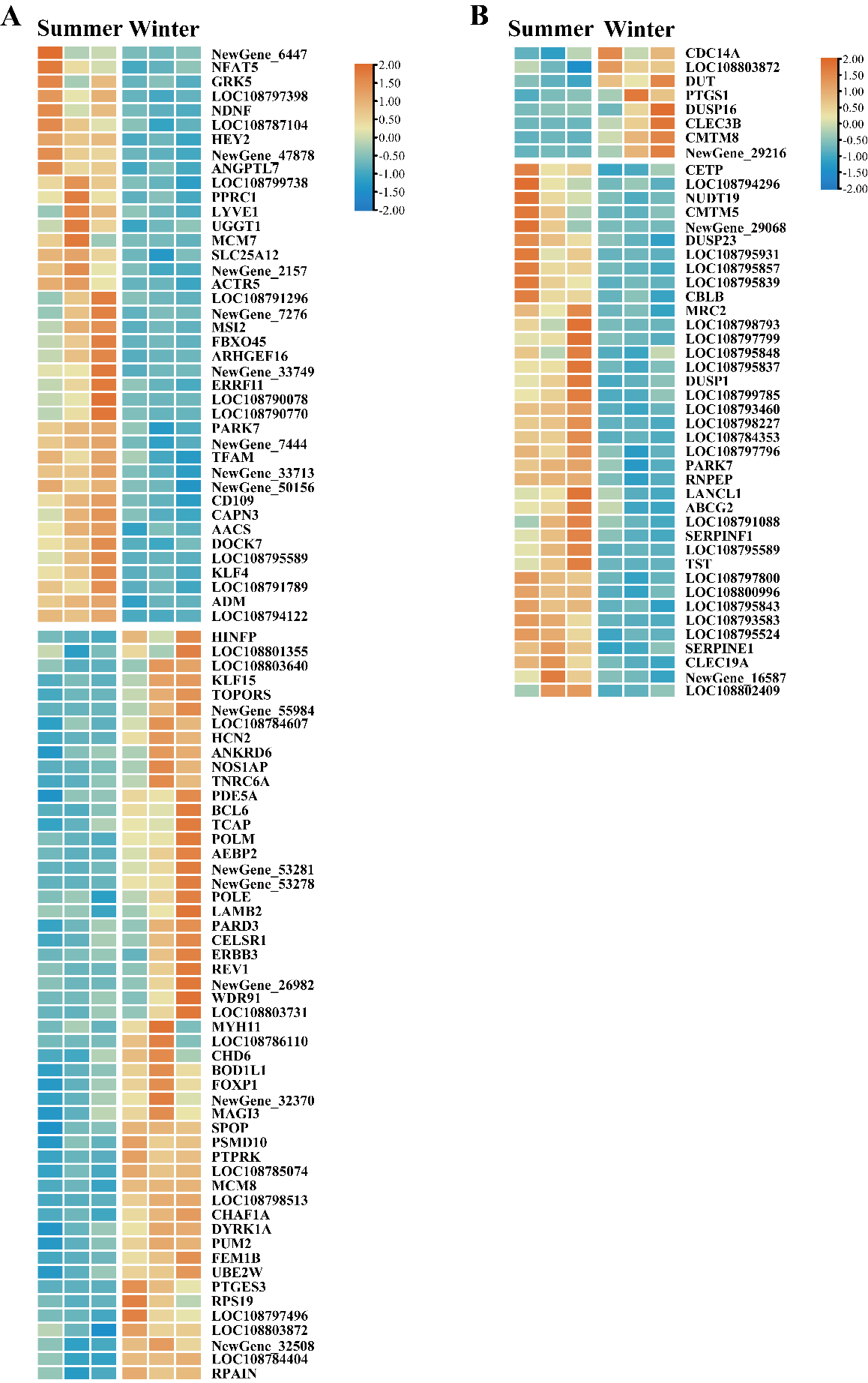


Supplementary Fig. 2. Heat map showing differentially expressed genes in skeletal muscles involved in “response to stress” (A) and “defense mechanisms” (B), respectively. Red color indicates highly expressed genes, and blue indicates low expressed genes.


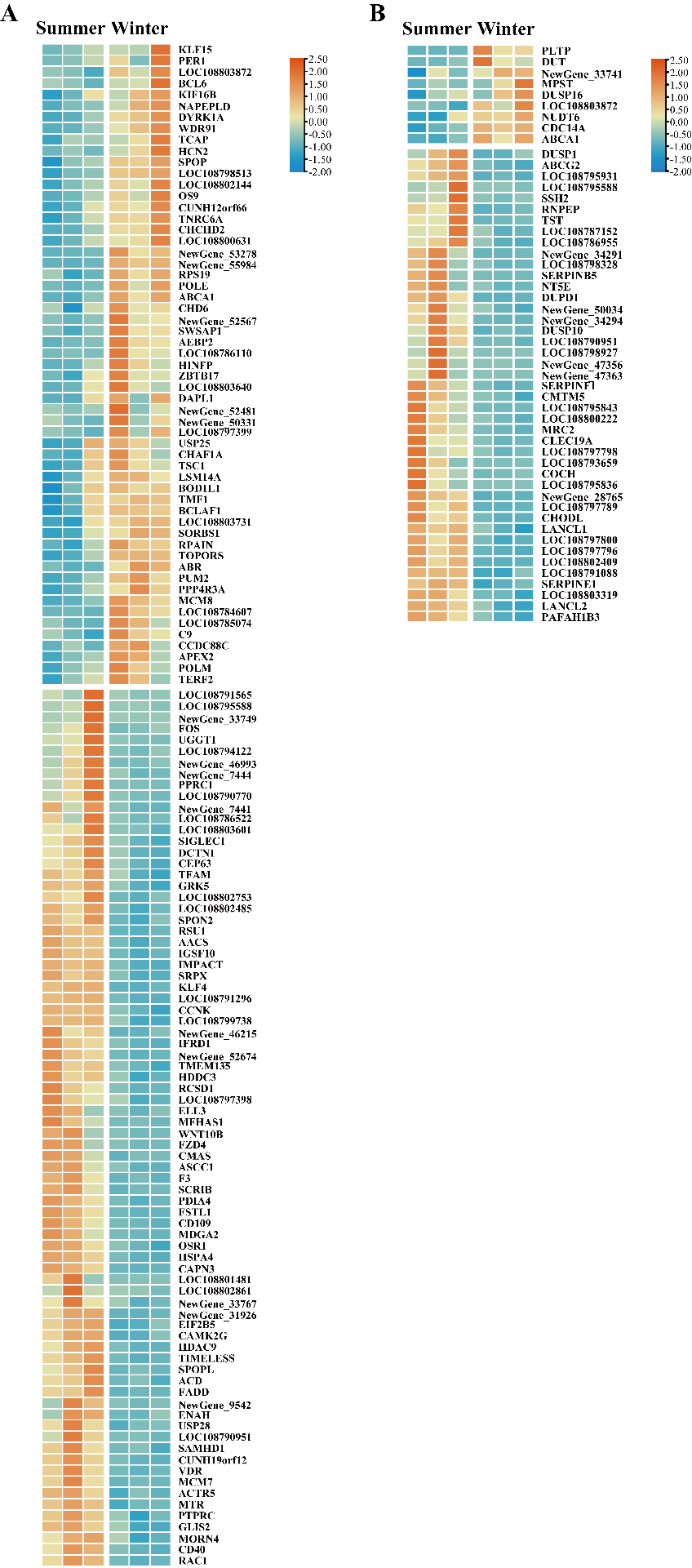

Supplement: Supplementary file 1 — Supplementary Material 1 [file 12864_2024_10357_MOESM1_ESM.docx]
